# Gut microbiome dynamics in autism: a prospective nested case–control study demonstrates microbial-clinical associations following rehabilitation interventions

**DOI:** 10.3389/fnins.2026.1820904

**Published:** 2026-06-08

**Authors:** Juanjuan Chen, Jinying Wei, Tianyu Liu, Jiayi Chen, Yanhan Yuan, Feng Zhang, Jinping Zhang

**Affiliations:** 1 Pediatrics, Shanghai Sixth People's Hospital Affiliated to Shanghai Jiao Tong University School of Medicine, Shanghai, China; 2 Guangxi Key Laboratory of Germplasm Innovation and Utilization of Specialty Commercial Crops in North Guangxi, Guangxi Academy of Specialty Crops, Guilin, China

**Keywords:** autism spectrum disorder, growth and development, gut microbiome, rehabilitation training, sleep problem

## Abstract

**Background:**

Children with autism spectrum disorder (ASD) commonly exhibit gut microbiota dysbiosis and metabolic abnormalities, yet the mechanisms linking these changes to clinical symptoms remain unclear.

**Objective:**

This study employed a nested case–control design and multi-omics approaches to evaluate the effects of rehabilitation intervention on clinical symptoms and gut microbiota in children with ASD, identify distinct microbial-metabolic signatures, and explore their mechanistic links with sleep disorders and developmental abilities.

**Methods:**

Within a prospectively established pediatric cohort (*n* = 45), we implemented a nested case–control design including 26 ASD children (18 males, 8 females; mean age 61.79 ± 11.15 months) and 19 age- and sex-matched healthy controls. All ASD participants received standardized rehabilitation therapy (2 h/day, 5 days/week for 6 months) comprising occupational therapy and cognitive-linguistic training. Primary outcomes included comprehensive clinical assessments [Griffiths Development Scales-Chinese (GDS-C), Children’s Sleep Habits Questionnaire (CSHQ), Autism Behavior Checklist (ABC), Childhood Autism Rating Scale (CARS)] and longitudinal multi-omics analysis (metagenomic sequencing and LC–MS-based metabolomics). Association analyses were performed with FDR correction (*q* < 0.05).

**Results:**

Following the 6-month rehabilitation intervention, significant clinical improvements were observed in sleep quality (CSHQ total and subscores) and developmental performance (GDS-C). Multi-omics profiling revealed distinct biological signatures in ASD children compared to healthy controls, characterized by elevated *Intestinibacter_bartlettii* and reduced levels of ornithine and siderophore nonribosomal peptide biosynthesis. Crucially, correlation analysis demonstrated that, after FDR correction, ornithine levels were significantly positively correlated with multiple GDS-C developmental domains, while tyrosine was associated with parasomnias. These findings establish a potential mechanistic link where amino acid metabolism connects gut microbial shifts to clinical phenotypes.

**Conclusion:**

This study demonstrates that rehabilitation intervention synchronously ameliorates clinical symptoms and modulates the gut-metabolic profile in ASD. The identified associations between specific metabolites (ornithine and tyrosine) and clinical outcomes suggest a metabolic mechanism underlying the gut-brain axis, highlighting the potential of these metabolites as biomarkers for therapeutic monitoring. Further large-scale studies are needed to validate these findings.

## 1 Introduction

Autism spectrum disorder (ASD) is a heterogeneous neurodevelopmental disorder characterized by core symptoms including social communication impairments, restricted interests, and repetitive stereotyped behaviors (Bundgaard-Nielsen et al., 2023). In recent years, the global prevalence of ASD has shown an increasing trend, posing a significant public health challenge (Maenner et al., 2023; Zhou et al., 2020). The precise etiology of ASD remains unclear. While genetic factors play a role, the “missing heritability” suggests the crucial involvement of environmental factors and epigenetic regulation (Wiśniowiecka-Kowalnik and Nowakowska, 2019). Emerging evidence indicates that gut microbiota dysbiosis contributes to ASD pathogenesis through the microbiota-gut-brain axis (Liu et al., 2019; Taniya et al., 2022; Gyawali and Patra, 2019). The microbiota-gut-brain axis represents a bidirectional communication network involving neural, endocrine, and immune pathways (Lasheras et al., 2020). Comprising trillions of microorganisms, the gut microbiota plays a pivotal role in regulating this axis through metabolite production, immune modulation, and neurotransmitter synthesis (Mhanna et al., 2024; Hou et al., 2022). ASD patients typically exhibit altered gut microbiome composition, characterized by reduced microbial diversity and bacterial imbalance (Kurokawa et al., 2024).

Currently, rehabilitation training serves as the primary clinical intervention for ASD, though treatment efficacy shows significant individual variability. Existing research reveals critical gaps regarding the interplay between rehabilitation, gut microbiota, and clinical outcomes. While cross-sectional studies consistently report distinct microbial imbalances in ASD children, longitudinal intervention studies often fail to confirm causal relationships between specific microbial changes and symptom improvement. Furthermore, despite the high comorbidity of sleep disorders in ASD children (Hossain et al., 2020), research examining the cascading effects of the microbiota-sleep-neurodevelopment axis remains limited.

To address these gaps, we hypothesize that rehabilitation training may improve core ASD symptoms and comorbidities by reshaping gut microbial metabolic balance. To test this hypothesis, we designed a prospective nested case–control study employing metagenomics and untargeted metabolomics techniques, combined with assessment tools including GDS-C, CSHQ, ABC, and CARS. This study aims to investigate the longitudinal shifts in gut microbiota and metabolites in ASD children following intervention and to determine their specific associations with sleep health and developmental abilities.

## 2 Materials and methods

### 2.1 Subjects

**Figure 1:**
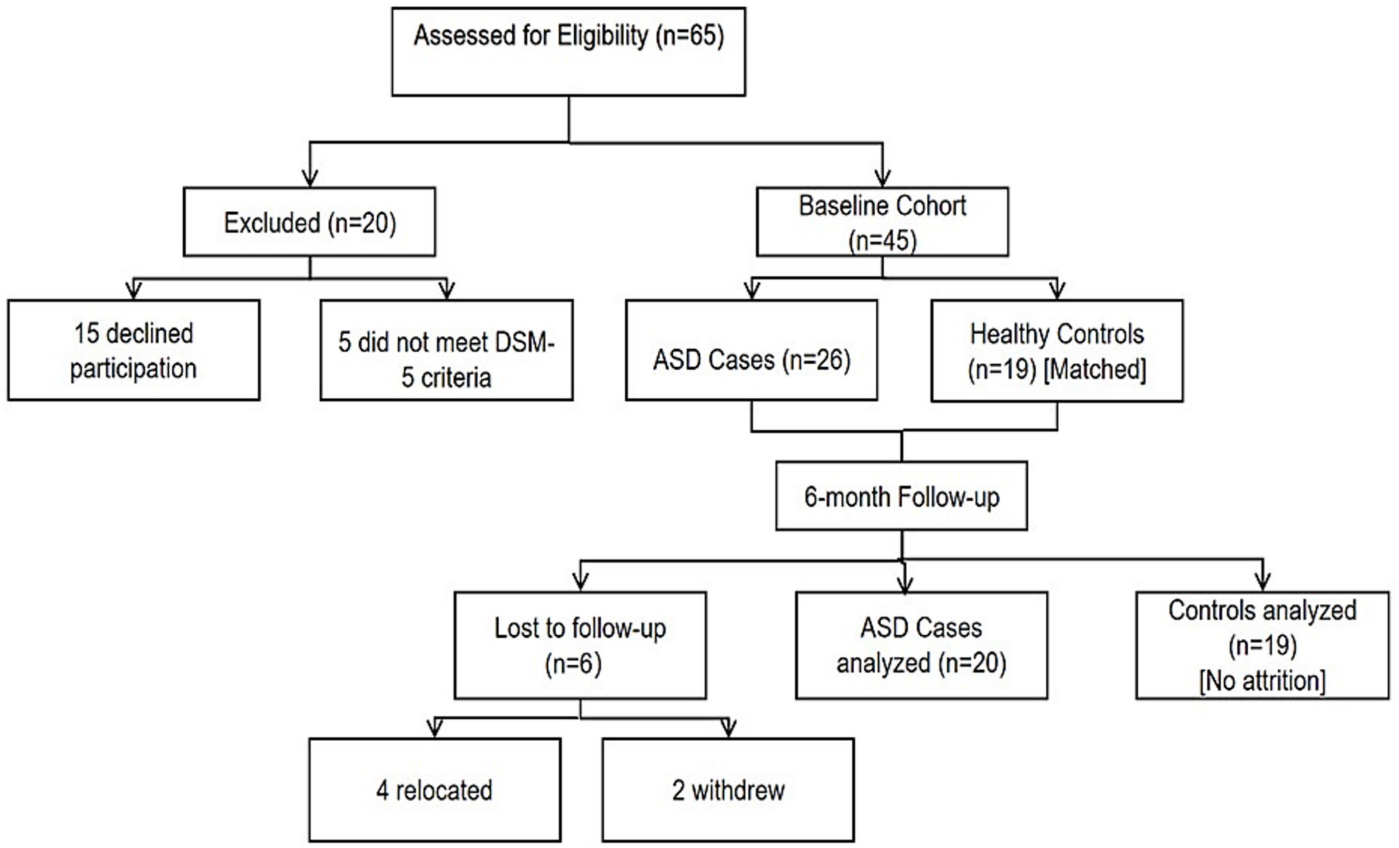
CONSORT flow diagram.

This nested case–control study was conducted within a pediatric health follow-up cohort established at the Lingang Pediatric Center of Shanghai Sixth People’s Hospital from January to December 2023. As a real-world study, the protocol was approved by the Ethics Committee of Shanghai Sixth People’s Hospital (Approval No. 2021–276). Written informed consent was obtained from all eligible parents or guardians prior to enrollment. Initial Cohort: From 65 initially screened potential participants, 20 were excluded (15 due to non-compliance, 5 not meeting diagnostic criteria), resulting in a baseline cohort of 45 children. Case Group: Twenty-six children were diagnosed with ASD by two board-certified pediatricians according to DSM-5 criteria (American Psychiatric Association, 2013). After 6 months of follow-up, 20 cases completed the final analysis (6 lost to follow-up: 4 relocated, 2 withdrew consent). Control Group: Nineteen matched controls were selected from children undergoing routine health examinations at the same clinic during the same period. Matching criteria included age (±6 months), sex, height, and weight (Table 1). Biological sample collection for controls was synchronized with the baseline assessment of the case group. The CONSORT flow diagram is shown in Figure 1.

**Table 1:** General clinical data.

Variable	ASD group (*n* = 26)	asd group (*n* = 20)	C group (*n* = 19)	Statistic	*p*
Age (months)	61.79 ± 11.15	66.87 ± 11.19	64.98 ± 13.35	1.09	0.34
Gender					
Male (%)	18 (69%)	14 (70%)	14 (74%)	0.82	0.66
Female (%)	8 (31%)	6 (30%)	5 (26%)		
Weight (Kg)	18.18 ± 2.11	19.68 ± 2.25	19.93 ± 4.06	2.56	0.09
Height (cm)	109.08 ± 5.47	112.19 ± 5.87	111.18 ± 7.55	1.49	0.23

Inclusion Criteria: (1) Age between 3 and 8 years old, male or female; (2) No medications and foods that significantly affect the intestinal flora in the last 3 months, such as probiotics, prebiotics, antibiotics, and fecal transplantation therapy; (3) Normal weight and height corresponding to his/her age; (4) Unchangeble dietary habits within 6 months; (5) Voluntary participation in this study.

Exclusion criteria: (1) A high fever greater than or equal to 41 °C within 1 month prior to enrolment; (2) Severe malnutrition;(3) use of antibiotics within 1 month prior to sample collection; (4) Special diets such as ketogenic diets; (5) Psychiatric disorders other than autism; (6) Immunodeficiency disease; (7) severe gastrointestinal disorders, such as intestinal obstruction; (8) Participation in other clinical studies.

### 2.2 Assessment scale

The Childhood Autism Rating Scale (CARS) (Moon et al., 2019) was used by professional child psychologists to assess the severity of ASD symptoms in children already diagnosed based on DSM-5 criteria. According to the standard scoring criteria, a composite score of less than 30 indicates the absence of autism characteristics. The higher the score, the more severe the ASD symptoms. Specifically, a total score of 36 or more, with at least five indicators rated greater than 3, identifies cases of severe autism, while a score between 30 and 36 is considered mild to moderate autism.

The Autism Behavior Checklist (Volkmar et al., 1988) is used to assess abnormalities in the areas of perception, movement, emotion, and language in children with autism. The guardians were required to live with the children for at least 6 months to ensure the accuracy of their observations of the children. If the total score is 31 or more, autism may be suspected; if the total score is 67 or more, the risk of autism is greater.

The growth and development of children with autism was assessed using the Griffiths Development Scales-Chinese Edition (Li et al., 2020), which is available for children aged 0–2 years and 0–8 years. The GDS-C for 0–2 years has the following 5 sections: locomotor (A1), personal-social (B1), hearing and language (C1), hand-eye coordination (D1), and performance (E1), and for 0–8 years, the practical reasoning section (F1) was added to this version. Since children with ASD scored very low in the practical reasoning section (F1) of this study, there were only 5 domains.

The Children’s Sleep Habits Questionnaire (Wang et al., 2013) was used to assess sleep disorders in children with autism. The Chinese version of this instrument contains 45 questions covering eight specific sleep problems in the areas of bedtime resistance (S1), sleep anxiety (S2), sleep duration (S3), sleep disordered breathing (S4), parasomnias (S5), daytime sleepiness (S6), night wakings (S7), and sleep onset delay (S8). When the CSHQ score exceeds 41, it means that there is a sleep problem.

### 2.3 Rehabilitation methods

#### 2.3.1 Individualized rehabilitation protocol

To control for intervention dosage as a confounding variable, the structural framework of the rehabilitation program was standardized across all participants. However, the specific training content and difficulty levels within each module were individualized based on the GDS-C developmental quotient assessment results. All participating therapists held nationally certified rehabilitation therapist qualifications and had at least 5 years of clinical experience in ASD intervention. Prior to implementation, the therapy team underwent a 2-week standardized training program to ensure intervention fidelity.

#### 2.3.2 Structured rehabilitation framework

##### 2.3.2.1 The protocol comprised four core therapeutic modules

A. Motor Therapy.

Aimed at improving gross motor function and coordination:

- Balance training (single-leg stance, balance beam walking).
- Ball skills training (throwing/catching, kicking).
- Fundamental fitness training (obstacle running, jumping).

B. Occupational Therapy.

Focused on enhancing activities of daily living (ADL):

- Fine motor training (beading, block building).
- Basic self-care skills (independent feeding, toileting, dressing).
- Utensil use training (cutlery, stationery).

C. Sensory Integration Therapy.

Targeted sensory processing and environmental adaptation:

- Vestibular training (swinging, sliding).
- Proprioceptive training (trampoline, climbing).
- Tactile training (weighted vests, texture brushes).

D. Cognitive-Linguistic Training.

Emphasized language comprehension and expression:

- Auditory comprehension (following verbal instructions).
- Expressive language (echolalia, picture description).
- Social communication (conversational turn-taking, emotional labeling).

##### 2.3.2.2 Intervention parameters

- Session duration: 30 min per module.
- Daily schedule: 2 modules each in morning/afternoon sessions.
- Inter-module interval: 10 min.
- Frequency: 5 days/week (Monday–Friday).
- Total duration: 6 months.
- Delivery mode: One-on-one individualized training.

### 2.4 Fecal sample collection

Fecal samples were collected at two time points: before the initiation of the rehabilitation intervention (baseline) and immediately after the 6-month intervention period (post-intervention). To minimize the influence of diurnal variation on gut microbiota composition, all participants were instructed to collect samples in the morning (between 7:00 a.m. and 9:00 a.m.). Given the young age of the participants (3–8 years), parents or caregivers assisted in the collection process under the guidance of research staff. Fresh fecal specimens were collected in sterile containers, ensuring no contamination by urine. The exact collection time was recorded, and parents transported the samples on ice to the hospital within 2 h. Upon arrival, the specimens were homogenized and immediately stored at −80 °C until analysis. For Macrogenetic sequencing, approximately 200 mg of fecal material was used, while 300 mg was used for untargeted metabolomics analysis.

### 2.5 Metagenomic profiling

#### 2.5.1 Deoxyribonucleic acid extraction and sequencing

An ultrasonic crusher was utilized to randomly break the qualified deoxyribonucleic acid (DNA) samples into fragments of around 350 bp in length. The whole library preparation was completed via end repair, the addition of 3′ end A and sequencing adapters, purification, fragment selection, polymerase chain reaction (PCR) amplification and other steps. After the completion of library construction, the quantitative PCR (qPCR) method was employed to accurately quantify the effective concentration of the library (library effective concentration >3 nM) to ensure its quality for further sequencing. A 2 × 150 bp paired-end protocol was used to sequence metagenomic DNA on Illumina HiSeq.

#### 2.5.2 Sequencing data quality control

Trimmomatic (v_0.39) was applied to remove low-quality sequences. The quality control of sequencing reads was conducted to remove low-quality reads and trim low-quality bases. KneadData was adopted to remove the contamination sequence from human DNA. Before and after removal, FastqQC was used to examine sequence quality.

#### 2.5.3 Taxonomy annotation

Host-filtered microbial reads underwent classification against viral, bacterial, archaeal, fungal and human genomes by use of Kraken2 on a reference database. With bacteria, fungi, archaea and virus sequences, the reference database was constructed from NCBI nucleotide and RefSeq database. After that, the classification report was used by Bracken for estimating species abundance, which provided estimated reads per species.

#### 2.5.4 Functional annotation

Functional analysis was made using HUMAnN2 based on the UniRef90 database and annotated by the Kyoto Encyclopedia of Genes and Genomes (KEGG) database to get KEGG ontology (KO) and pathway level profile per sample.

### 2.6 Metabolite profiling

#### 2.6.1 Sample preparation

Liquid nitrogen was leveraged to ground feces (100 mg) individually, and prechilled 80% methanol was used to resuspend the homogenate by well vortex. The samples were subjected to 5-min incubation on ice and then 20-min centrifugation at 15,000 g at the temperature of 4 °C. Liquid chromatography-mass spectrometry (LC–MS) grade water was utilized to dilute some of the supernatants to the final concentration with 53% methanol, followed by the transfer of the samples to a fresh Eppendorf tube and their 20-min centrifugation at 15,000 g at 4 °C. At last, the LC–MS/MS system was injected with the supernatant for analysis.

#### 2.6.2 LC–MS analyses

A Vanquish UHPLC system (Thermo Fisher, Germany), along with an Orbitrap Q Exactive™ HF or Orbitrap Q Exactive™ HF-X mass spectrometer (Thermo Fisher, Germany) was used to perform ultra-high performance liquid chromatography (UHPLC)-MS/MS analyses. A 17-min linear gradient was utilized to inject the samples onto a Hypersil GOLD column (100 × 2.1 mm, 1.9 μm) at a flow rate of 0.2 mL/min. The eluents for positive and negative polarity modes were eluents A (0.1% FA in water) and B (methanol), and eluents A (5 mM ammonium acetate, pH 9.0) and B (methanol), respectively. Below was the set solvent gradient: 2% B, 1.5 min; 2–85% B, 3 min; 85–100% B, 10 min; 100–2% B, 10.1 min; 2% B, 12 min. Q Exactive™ HF mass spectrometer was operated in the positive/negative polarity mode. The spray voltage was 3.5 kV; the capillary temperature was 320 °C; the sheath gas flow rate was 35 psi; the aux gas flow rate was 10 L/min; the S-lens RF level was 60; the Aux gas heater temperature was 350 °C.

#### 2.6.3 Data processing and metabolite identification, and statistical analysis

Compound Discoverer 3.3 (CD3.3, Thermo Fisher) was adopted to process the raw data files produced by UHPLC–MS/MS to perform peak alignment, picking, and quantification. The main processing parameters were set as follows: peak area correction referenced to the first quality control (QC) sample, an actual mass tolerance of 5 ppm, and a signal intensity tolerance of 30%. Metabolite identification was performed by matching the accurate mass and MS/MS fragmentation patterns against the mzCloud, mzVault, and a custom MassList database. Annotation confidence was assigned according to the Metabolomics Standards Initiative (MSI) levels, with identifications primarily reaching MSI Level 2 (putative annotation based on spectral library matching).

A standardized data preprocessing pipeline was applied to the extracted peak intensity matrix prior to statistical analysis: (1) Filtering: Metabolic features with a missing rate >50% in any sample group were removed. (2) Imputation: Remaining missing values were imputed using the minimum value for each feature. (3) Normalization: To correct for variations in overall metabolite concentration, peak intensities were normalized by the median value of each sample. (4) Transformation and Scaling: The normalized data were subsequently log2-transformed and then auto-scaled (mean-centered and divided by the standard deviation per metabolite) to achieve a normal distribution and comparable variance across all features for downstream multivariate analysis.

Differential metabolite selection was based on the following consecutive criteria applied to the preprocessed data: (1) Multivariate Analysis: Variable Importance in Projection (VIP) scores were derived from an Orthogonal Partial Least Squares-Discriminant Analysis (OPLS-DA) model, and metabolites with a VIP score >1.0 were retained for further univariate testing. (2) Univariate Significance: The non-parametric Kruskal-Wallis test was used to assess inter-group differences, retaining metabolites with a *p*-value < 0.05. (3) Fold Change Threshold: The absolute log2 Fold Change (FC) >0.263 (equivalent to a linear FC > 1.2 or <0.833) was applied to select metabolites with biologically relevant magnitude of change. (4) Multiple Testing Correction: The Benjamini–Hochberg method was applied to control the False Discovery Rate (FDR), and only metabolites with an FDR-adjusted *p*-value (*q*-value) < 0.05 were considered statistically significant.

The normalized and scaled data were used for principal component analysis (PCA) and correlation analyses with species abundance. The statistical software R (version 4.3.1) was applied to perform all statistical analyses.

### 2.7 Statistical methods

Statistical analyses were conducted as follows: Data are presented as Mean ± SEM (x̄ ± SEM), median (interquartile range); Demographic data were analyzed using one-way ANOVA or chi-square tests; pre- and post-rehabilitation comparisons were performed using the Wilcoxon signed-rank test; Intergroup differences in microbial/metabolite profiles were assessed by the Kruskal-Wallis test; correlations involving GDS-C, CSHQ, and differential species were evaluated using Pearson’s method; all figures were generated using R; and the significance threshold was set at *p* < 0.05.

Student’s *T*-test was used for analyzing the alpha (*α*) diversity between groups, and Bray–Curtis dissimilarity was employed to calculate beta (*β*) diversity. The impact of phenotype on taxon/metabolite profiles was evaluated by performing permutational multivariate analysis of variance (PERMANOVA) utilizing the “adonis” function in the R Vegan package. For metagenomic and metabolomic data, the Benjamini–Hochberg method was applied for multiple testing correction, with a false discovery rate (FDR) threshold of <0.05 maintained across all high-throughput analyses.

## 3 Results

### 3.1 Clinical characteristics and changes in scales after rehabilitation

After strict inclusion and exclusion criteria were applied, a total of 45 children were recruited in this study (Table 1). They were divided into three groups: the ASD group, who were at the baseline, the asd group, who had received a six-month rehabilitation, and the control group, who were healthy. There were no statistically significant differences in age, gender, weight, and height between the ASD, asd, and C groups (*p* > 0.05).

After 6 months of rehabilitation, scores on both the CARS and ABC scales, as well as the proportion of children with CARS scores >36, showed a decreasing trend. However, these changes were not statistically significant (*p* = 0.615, *p* = 0.053), indicating that the rehabilitation intervention did not produce a significant therapeutic effect on the core symptoms of children with ASD (Table 2).

**Table 2:** Data analysis of clinical scales.

Variable	ASD group (*n* = 26)	asd group (*n* = 20)	*p*
CARS	37.50 (29.25–40.00)	34.00 (30.00–37.00)	0.615
CARS ≥ 36 (*n*, %)	15 (57.69%)	11 (55.00%)	Not tested
CARS < 36 (*n*, %)	11 (42.31%)	9 (45.00%)	Not tested
ABC total	65.50 (58.00–72.50)	59.50 (56.00–67.50)	0.053
CSHQ total	71.50 (67.25–75.75)	64.50 (61.00–66.50)	<0.001
CSHQ subscale scores			
S1 bedtime resistance	11.50 (10.00–12.00)	11.00 (10.00–12.00)	0.459
S2 sleep anxiety	8.00 (6.75–9.00)	8.00 (6.25–8.00)	0.238
S3 sleep duration	5.00 (5.00–5.00)	4.00 (3.00–5.00)	0.01
S4 sleep disordered breathing	8.00 (7.00–9.00)	8.00 (7.00–9.00)	0.102
S5 parasomnias	18.00 (17.00–21.00)	18.00 (17.00–19.00)	0.096
S6 daytime sleepiness	16.00 (15.00–18.00)	13.00 (13.00–14.75)	0.002
S7 night wakings	8.00 (7.00–9.00)	5.50 (5.00–6.00)	<0.001
S8 sleep onset delay	2.00 (2.00–3.00)	2.00 (1.00–2.00)	0.007
GDS-C subscale scores			
Locomotor	48.00 (38.21–60.50)	52.00 (42.50–63.50)	0.122
Personal-social	45.00 (29.74–51.50)	49.00 (35.00–58.50)	0.121
Hearing and language	30.00 (13.45–38.50)	33.00 (18.43–41.50)	0.169
Hand-eye coordination	38.50 (33.50–48.50)	42.00 (28.50–50.00)	0.161
Performance	37.00 (22.58–47.34)	47.50 (38.50–61.00)	<0.001

Compared with the pre-rehabilitation ASD group.there were significant differences in CSHQ total (*p* < 0.001), sleep duration (*p* = 0.01), daytime sleepiness (*p* = 0.002), night wakings (*p* < 0.001), and sleep onset delay (*p* = 0.007). The result suggested that the rehabilitation intervention is more significant in CSHQ total sleep duration (S3) daytime sleepiness (S6), night wakings (S7), and sleep onset delay (S8) in children with ASD.

Since only 10 and 8 ASD children before and after rehabilitation were able to cooperate to complete the assessment of practical reasoning, and their scores were all lower than the baseline. Therefore, we only analyzed five fields: locomotor, personal-social, hearing and language, hand-eye coordination, and performance (Table 2). After rehabilitation, the scores of the children with ASD in 5 fields showed an upward trend, and there was a statistical difference in performance (*p* < 0.001). These results indicate that the rehabilitation intervention can improve sleep quality and enhance specific developmental domains, particularly the performance domain, in children with ASD.

### 3.2 Intestinal microbiota analysis of autistic and control subjects

**Figure 2:**
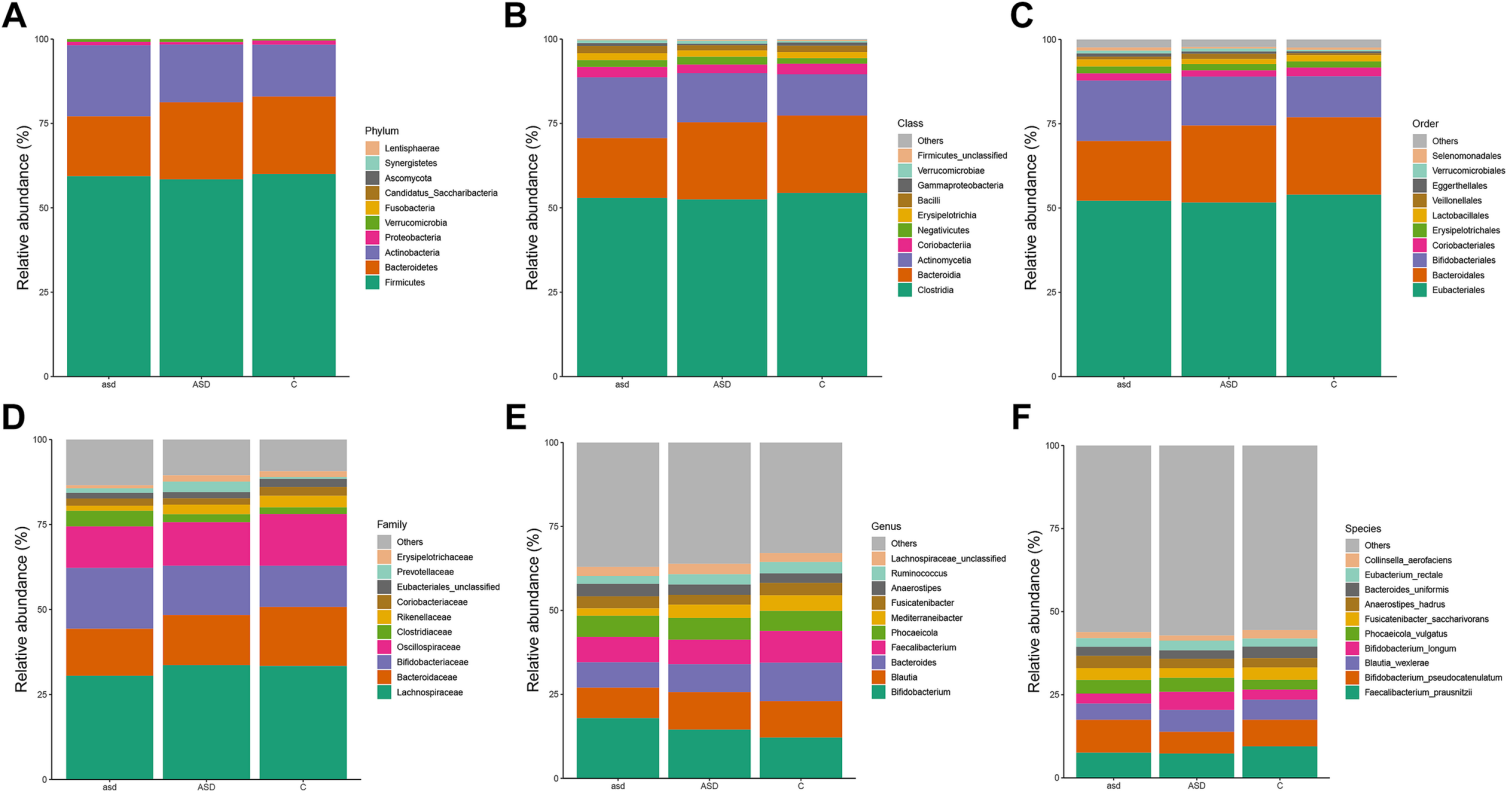
Relative abundance of asd, ASD, and C groups performed by the Kruskal–Wallis test: **(A)** The phylum level; **(B)** the class level; **(C)** the order level; **(D)** the family level; **(E)** the genus level; **(F)** the species level.

To determine the major species composition differences among the ASD group, the asd group, and the C group, the composition of the top 10 highest key microbial species was analyzed at various levels (Figure 2). K–W test results showed that there were no statistical differences among the 10 species with the highest abundance in the ASD group, asd group, and C group at phylum, class, order, family, genus, and species levels (*p* > 0.05).

### 3.3 Alpha diversity and Beta diversity analysis

**Figure 3:**
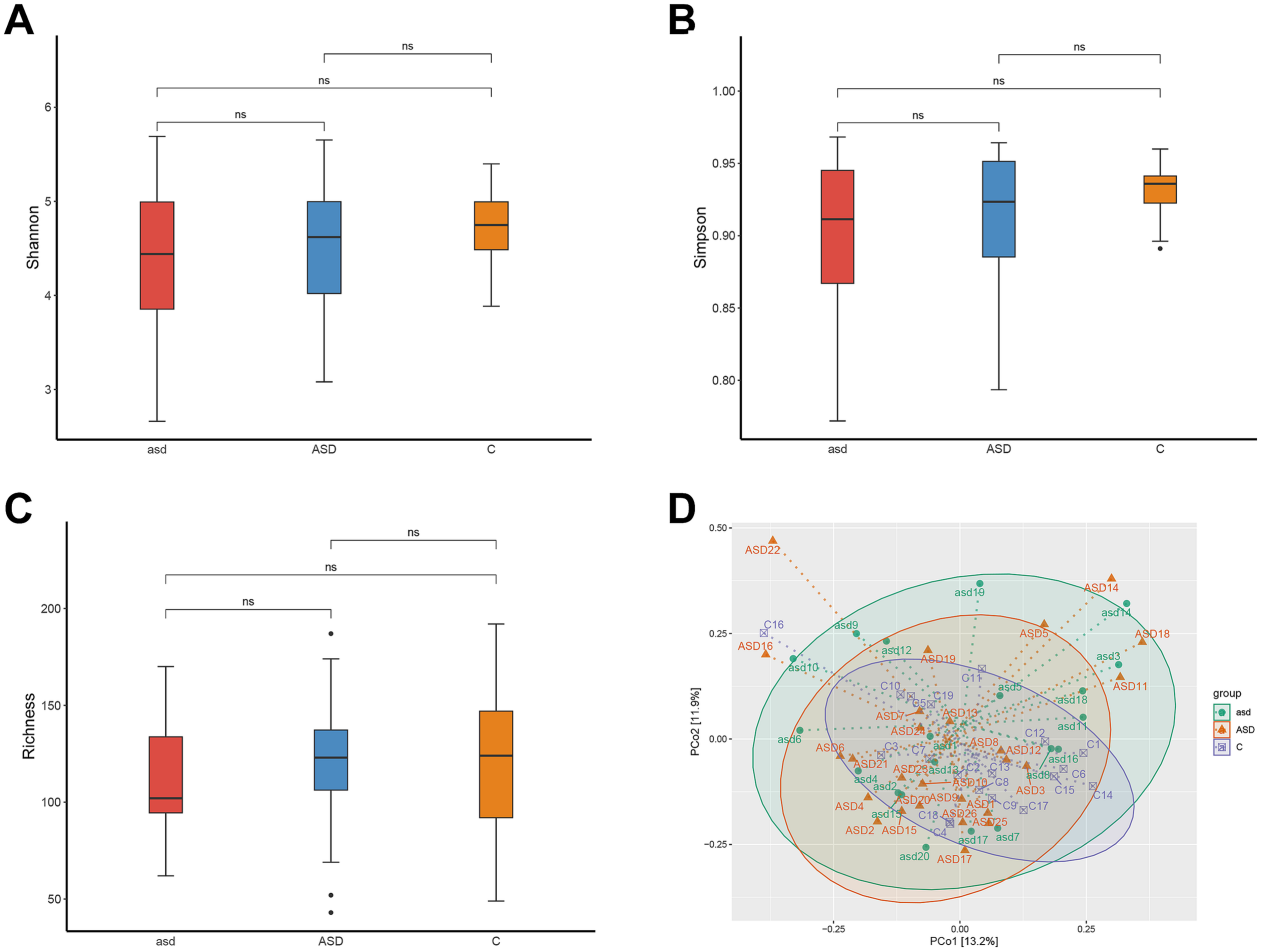
Alpha and beta diversity of asd, ASD, and C groups. **(A)** Shannon index. **(B)** Richness index. **(C)** Simpson index. **(D)** PcoA diagram.

Shannon index (Figure 3A), Richness index (Figure 3B), and Simpson index (Figure 3C) of the C group were higher than those of the ASD and asd groups. However, no statistically significant differences were observed (*p* > 0.05). The PcoA (Figure 3D) analysis showed no statistical difference between ASD, asd, and C groups, indicating that there was no significant difference in microbial species community structure among the three groups (*p* > 0.05). However, group C was exhibited a more compact distribution (lower dispersion; Figure 3D), suggesting higher intra-group homogeneity in microbial composition compared to the ASD and asd groups.

### 3.4 LefSe analysis

**Figure 4:**
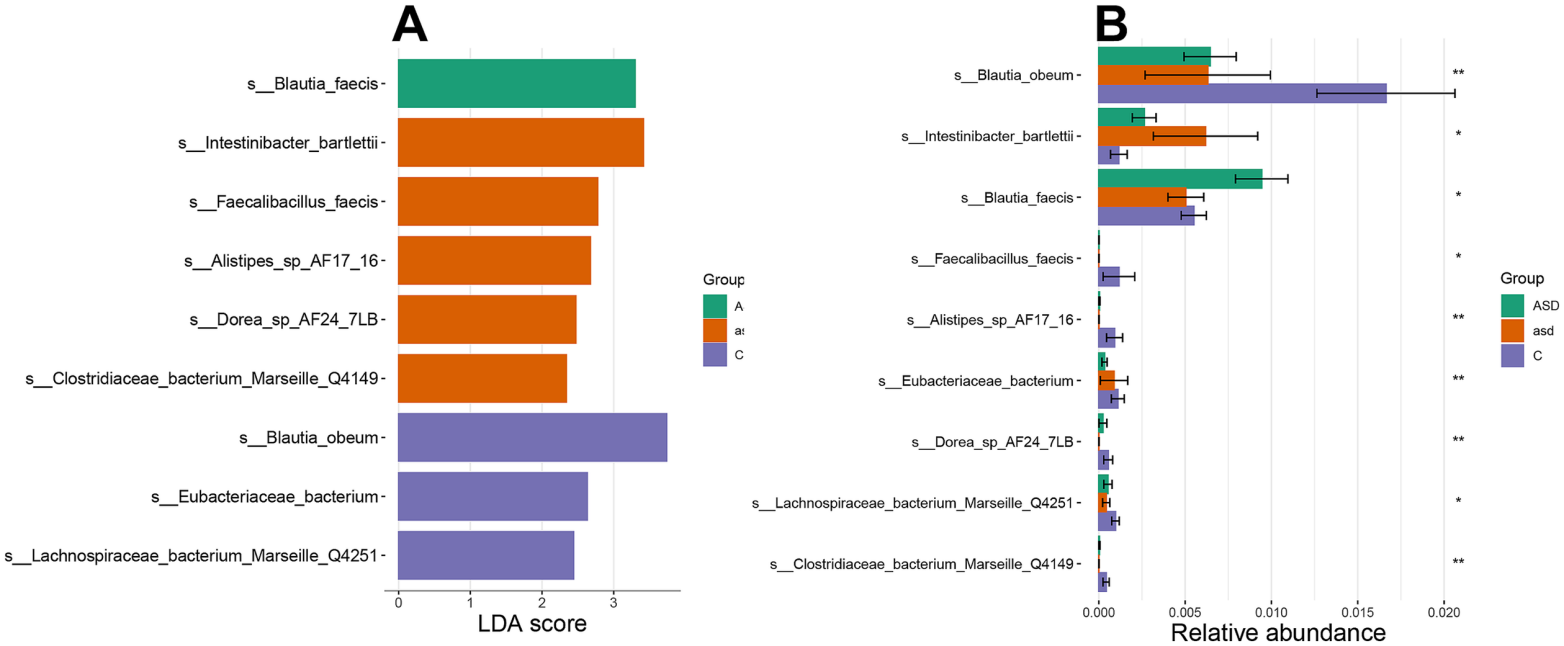
LEfSe analysis in ASD, asd, and C groups. **(A)** Selected differential microorganisms with linear discriminant analysis >2 at the level of phylum, class, order, family, genus, and species. **(B)** Average relative abundance of differential microorganisms. *p < 0.05, **p < 0.01.

LefSe analysis was used to search for different microorganisms at the phylum, class, order, family, genus, and species levels in different groups. The results (Figure 4) showed that there were 9 different species among the ASD group, asd group, and C group, and they were concentrated at the genus and species level. The abundance of *Intestinibacter_bartlettii* in group C was significantly lower than that in the ASD and asd groups (*p* < 0.05). The abundance of *Blautia_obeum, Dorea_sp_AF24_7LB, and Lachnospiraceae_bacterium_Marseille_Q4251* in the C group was significantly higher than that in the ASD and asd groups (*p* < 0.05). *Blautia_obeum, Dorea_sp_AF24_7LB, and Lachnospiraceae_bacterium_Marseille_Q4251* belong to the *Lachnospiraceae*.

### 3.5 Differential metabolite analysis

**Figure 5:**
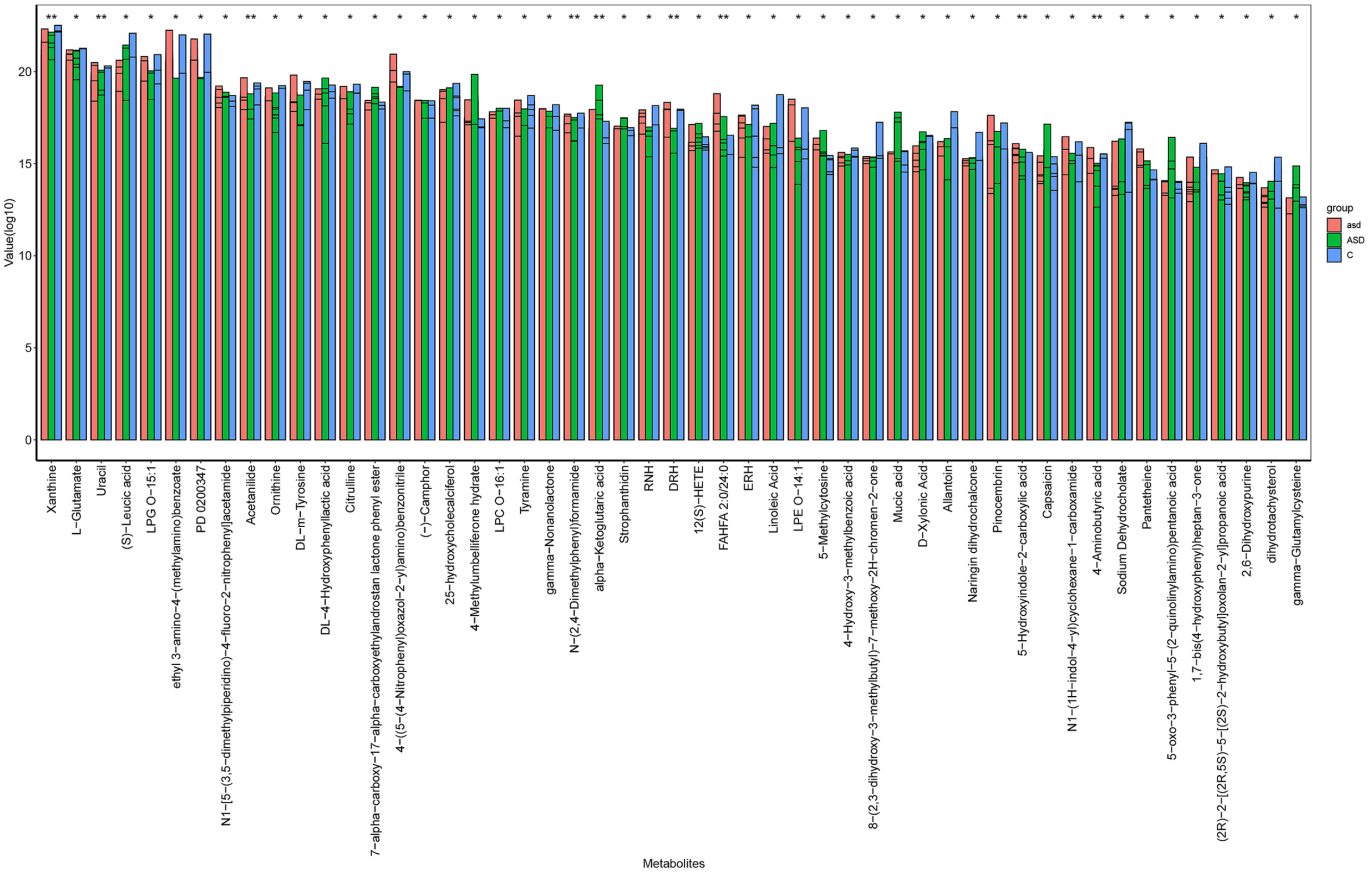
Average relative abundance bar chart of differential metabolites in the ASD, asd, and C group. *p < 0.05, **p < 0.01.

To identify major differentiating metabolites between groups, we used the Variable Importance in Projection (VIP) values from the OPLS-DA model combined with the Kruskal-Wallis test. The selection criteria were strictly defined as VIP > 1.0 and *p* < 0.05. Furthermore, to ensure statistical rigor, *p*-values were corrected using the False Discovery Rate (FDR), and a fold-change (FC) threshold was applied. Metabolite identification was performed based on Metabolomics Standards Initiative (MSI) level 2, utilizing database matching with authentic standards. Consequently, a total of 51 differential metabolites were identified (Figure 5). Ornithine and Tyrosine are amino acids that have important biological functions. Ornithine levels in the Pre-intervention and Post-intervention ASD groups were significantly lower than those in the Healthy Control group (*p* < 0.05). Additionally, a significant difference in ornithine levels was observed between the Pre- and Post-intervention ASD groups (*p* < 0.05). After rehabilitation, the tyrosine levels in the Post-intervention ASD group significantly increased (*p* < 0.05). The results indicated that there were differences in ornithine and tyrosine not only between children with ASD and healthy subjects but also in response to the rehabilitation intervention.

### 3.6 KEGG pathway enrichment analysis

**Figure 6:**
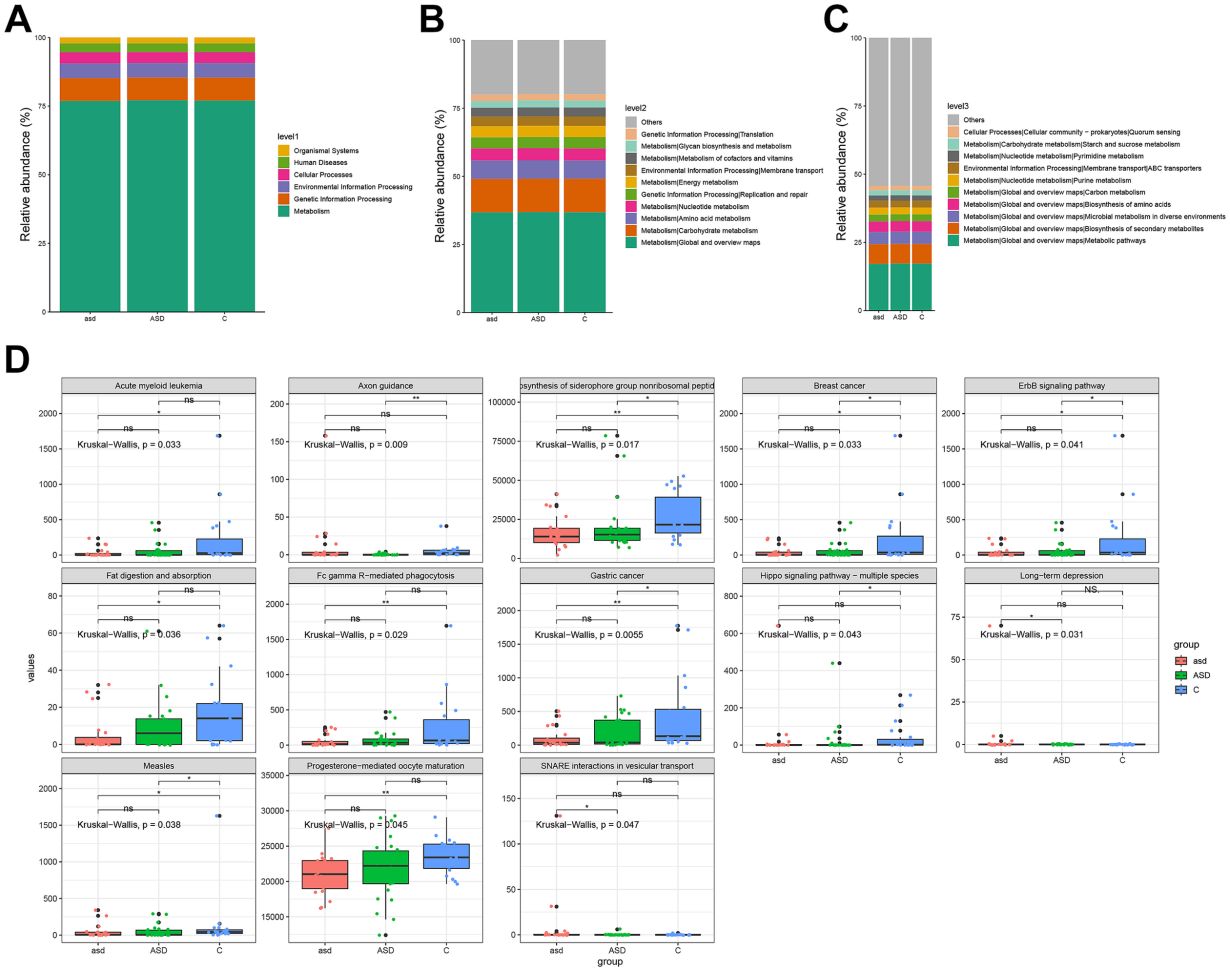
Relative abundance of KEGG metabolic pathways in ASD, asd, and C groups. **(A)** The primary level. **(B)** The secondary level. **(C)** The tertiary level. **(D)** The fourth level. *p < 0.05, **p < 0.01.

**Figure 7:**
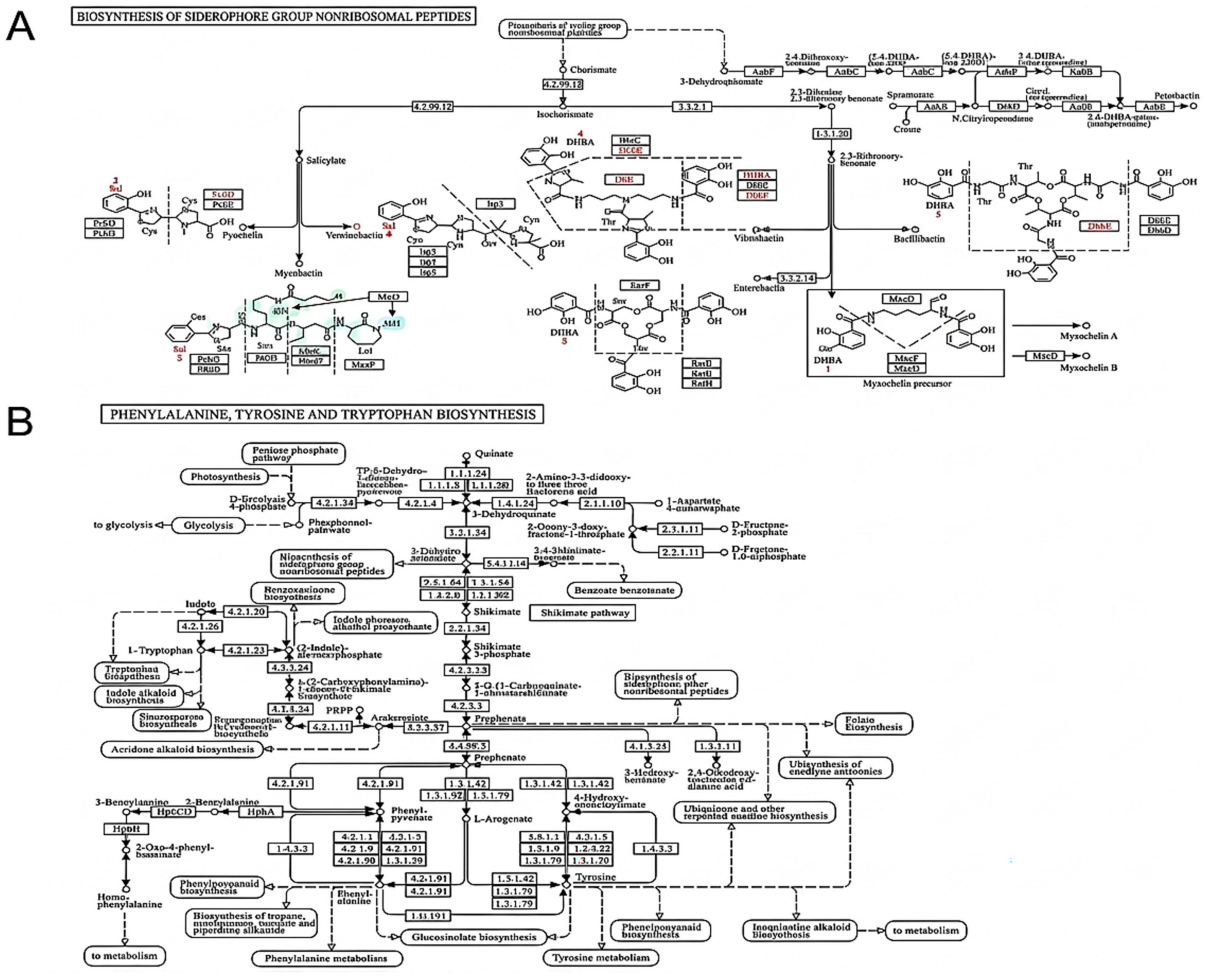
KEGG pathway. **(A)** Biosynthesis of siderophore group nonribosomal peptides. **(B)** Phenylalanine, tyrosine, and tryptophan biosynthesis.

Enrichment analysis of KEGG metabolic pathways at the primary, secondary, and tertiary levels in the Pre-intervention ASD, Post-intervention asd, and C groups (Figsures 6A–C) showed that there were no significant differences among the three groups. At the fourth level of the KEGG metabolic pathway (Figure 6D), there were a total of 13 differential metabolic pathways in the ASD, asd, and C groups, and all of them were statistically different (*p* < 0.05). Among them, the biosynthesis of the siderophore group nonribosomal peptides was the only pathway at the metabolic level, and therefore was the pathway we focused on. The results showed that the biosynthesis of the siderophore group nonribosomal peptides was significantly lower in the ASD and asd groups than in the C group (*p* < 0.05). The KEGG result showed that the biosynthesis of the siderophore group nonribosomal peptides pathway interacted with the synthesis pathways of phenylalanine, tyrosine, and tryptophan (Figures 7A,B).

### 3.7 Correlation analysis of GDS-C/CSHQ scales and differential species/metabolites

**Figure 8:**
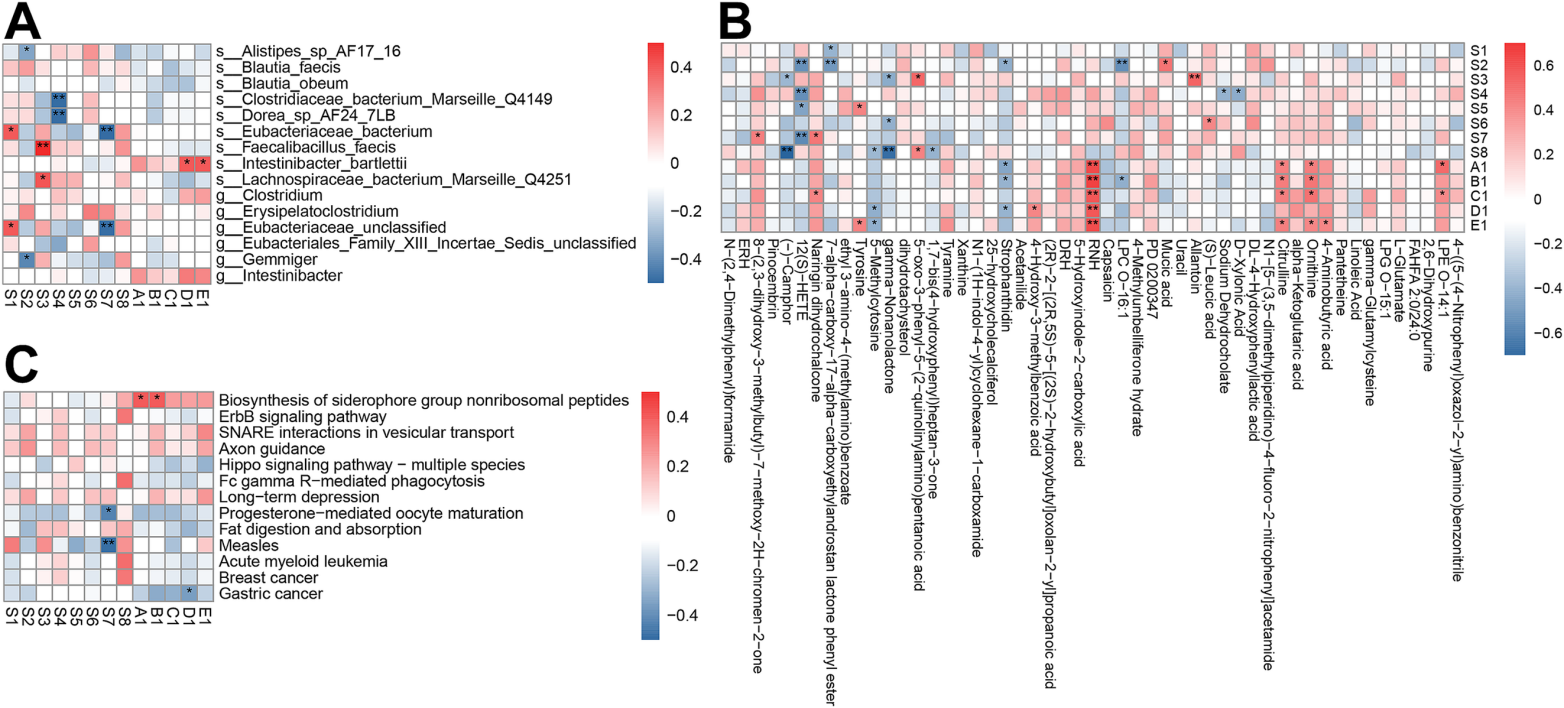
Correlation heatmap with Pearson correlation analysis. **(A)** GDS-C/CSHQ scale and differential microorganisms. **(B)** GDS-C/CSHQ scale and differential metabolites. **(C)** GDS-C/CSHQ scale and differential metabolic pathways. *p < 0.05, **p < 0.01.

To explore the relationship between development, sleep disorders, and intestinal flora in children with ASD, this analysis utilized data from the combined dataset of pre-intervention and post-intervention samples. Figure 8A showed the correlation analysis between 5 domains of the GDS-C scale, 8 dimensions of the CSHQ scale, and 15 different species. The results showed that there was a significant positive correlation between *I. bartlettii* and hand-eye coordination (D1; *r* = 0.26, *p* < 0.05) and performance (E1) (*r* = 0.23, *p* < 0.05). There were significantly positive relationships (*p* < 0.05) between bedtime resistance (S1) and *Eubacteriaceae bacterium* (*r* = 0.32), sleep duration (S3) and *Faecalibacillus_faecis* (*r* = 0.42), sleep duration (S3) and *Lachnospiraceae bacterium Marseille Q4251* (*r* = 0.33). There were significantly negative relationships (*p* < 0.05) between sleep anxiety (S2) and *Alistipes_ sp_ AF17_16* (*r* = −0.29), sleep disordered breathing (S4) and *Clostridiaceae bacterium Marseille Q4149* (*r* = −0.41), sleep disordered breathing (S4) and *Dorea* sp. *AF24 7LB* (*r* = −0.41), night wakings (S7) and *Eubacteriaceae bacterium* (*r* = −0.42). *Lachnospiraceae_bacterium_Marseille_Q4251*, *Clostridiaceae_bacterium_Marseille_Q4149*, *Dorea_ sp_AF24_7LB* belong to the Lachnospiraceae.

To explore the relationship between growth, development and sleep problems in children with ASD and intestinal metabolites, we conducted a correlation analysis on 5 domains of the GDS-C scale and 8 dimensions of the CSHQ scale and differential metabolites, as shown in Figure 7B. In the correlation analysis between the GDS-C scale and differential metabolites, locomotor (A1), personal society (B1), hearing and language (C1), hand-eye coordination (D1) and performance (E1) were significantly correlated with 5, 5, 5, 4, and 6 differential metabolites, respectively (*p* < 0.05). There were significant positive correlations between Ornithine and locomotor (A1) (*r* = 0.35), personal society (B1) (*r* = 0.38), hearing and language (C1) (*r* = 0.39), and performance (E1) (*r* = 0.33), and there were significant positive correlations between tyrosine and performance (E1) (*r* = 0.01). The results showed that the growth and development of children with ASD were significantly correlated with intestinal metabolites.

In the correlation analysis of the CSHQ scale and differential metabolites (Figure 8B), bedtime habits (S1), sleep anxiety (S2), sleep duration (S3), sleep disapnea (S4), parasomnia (S5), daytime sleepiness (S6), night wakefulness (S7) and sleep latency (S8) were significantly correlated with 1, 5, 4, 3, 2, 2, 3, and 5 different metabolites, respectively (*p* < 0.05). There was a significant positive correlation between tyrosine and heteromorphic sleep (S5) (*r* = 0.13). The results showed a significant correlation between sleep and intestinal metabolites in children with ASD.

Correlation analyses of the GDS-C/CSHQ scale and differential metabolic pathways are shown in Figure 8C, which show that there were significant positive correlations between both locomotor (A1) (*r* = 0.14) and personal-social (B1) (*r* = 0.23) and the biosynthesis of the siderophore group nonribosomal peptides (*p* < 0.05).

## 4 Discussion

ASD is a complex neurodevelopmental disorder for which there is no treatment that can completely cure it because the cause has not yet been elucidated (Taniya et al., 2022). The pathogenesis of ASD is very complex and involves a variety of causes, such as genetics, maternal pregnancy medication, and gut microbiota (Lungba et al., 2020). In recent years, as research on gut microbiota continues, more and more evidence is identifying its link and mechanism with ASD and comorbidities. Rehabilitation intervention is currently the primary treatment modality for children with ASD. Locomotor, personal-social, hearing and language, hand-eye coordination, and performance (*p* < 0.05) of children with ASD tended to increase after rehabilitation, and there was a corresponding decrease in scores on the CARS and ABC scales. We found a total of nine differential species in the asd, ASD, and C groups. Researchers have identified *Intestinibacter bartlettii* (formerly *Clostridium bartlettii*) as a potentially pathogenic bacterium in fecal microbiota. Comparative analyses revealed a significantly higher abundance of *I. bartlettii* in children with ASD compared to typically developing controls in this research, in agreement with the study by Bojovic et al. (2020). Current research has identified several gut microbiota species associated with the autism spectrum disorder (ASD), including members of the *Clostridium* (Zhou et al., 2025). Furthermore, we found a significant positive correlation between *I. bartlettii* and hand-eye coordination and performance. Gut microbes affect brain development and cognitive function in children (Gao et al., 2020). *I. bartlettii* produces indolelactic acid and indoleacetic acid, both of which are important products of tryptophan metabolism. Research by Liu et al. has identified an association between *Intestinibacter_bartlettii* and 3-indoleacetic acid (IAA) levels (Liu et al., 2025). While a causal relationship remains unconfirmed, these findings suggest that elevated *I. bartlettii* LEVELS may influence brain function through neuroendocrine pathways. Abnormalities in tryptophan metabolism have been reported in both plasma and feces of children with ASD, and a correlation between altered tryptophan and serotonin concentrations and intestinal dysbiosis has been observed in patients with ASD (Bonham et al., 2023).

In terms of the association between GSD-C and metabolism, there were significant positive correlations between ornithine and locomotor, personal-social, hearing and language, and performance, and significant positive correlations between tyrosine and performance and parasomnias. In addition, we found that ornithine was significantly lower in children with ASD than in healthy children, and the arachidonic acid metabolic pathway was significantly up-regulated after rehabilitation, consistent with the findings of Wang et al. in plasma metabolites in children with ASD (Wang et al., 2022). In a metabolite analysis of plasma from children with ASD, low levels of ornithine were found in male children with ASD (Bugajska et al., 2017). Further studies have found ornithine to be associated with language levels and social behavior in children with ASD and may be a potential biomarker for children with ASD (Laue et al., 2020)^.^ The arachidonic acid metabolic pathway produces inflammatory cytokines, and elevation of the arachidonic acid pathway may lead to increased levels of inflammation in the bodies of children with autism (Wang et al., 2022).

To explore the potential association between changes in sleep and changes in developmental outcomes following the intervention, we performed a post-hoc correlation analysis. We calculated the change scores for CSHQ total score and GDS-C performance score for each child and assessed their relationship using Spearman‘s rank correlation due to the small sample size and non-normal distribution of the change scores. The analysis indicated a negative, but non-significant, correlation between the improvement in sleep quality and the improvement in developmental performance (*r* = −0.149, *p* = 0.531, *n* = 20). This suggests that within our cohort, the magnitude of sleep improvement was not linearly predictive of the magnitude of gain in this specific developmental domain.

We found that the biosynthesis of the siderophore group nonribosomal peptides in children with ASD is significantly lower than that of healthy control children. Furthermore, there was a significant positive correlation between the pathway and exercise and personal socialisation, and it interacted with the phenylalanine, tyrosine, and tryptophan synthesis pathways. Studies have demonstrated significantly reduced tyrosine levels in both fecal and serum samples from children with the autism spectrum disorder (ASD; Randazzo et al., 2023). In the current investigation, we observed a marked elevation in tyrosine concentrations following rehabilitation therapy in children with ASD. Fang et al. have elucidated the protective effects of L-tyrosine in attenuating ASD-like behavioral impairments in valproic acid (VPA)-induced mouse models of ASD. Through integrated multi-omics analyses, they further investigated the underlying mechanisms. The ameliorative effects of L-tyrosine on autism-like behaviors may be associated with modulations in key KEGG pathways—including neuroactive ligand-receptor interaction, L-tyrosine metabolism, GABAergic synapse, and dopaminergic synapse—at both metabolomic and mRNA expression levels (Fang et al., 2025). Phenylalanine, tyrosine, and tryptophan may play a key role in the pathogenesis of autism spectrum disorders (Eicher and Mohajeri, 2022). Plasma phenylalanine levels were significantly higher, and plasma tyrosine levels were significantly lower in ASD patients compared to healthy controls (Jennings and Basiri, 2022). The activity of tyrosine and phenylalanine biosynthetic pathways was significantly lower in the prefrontal cortex of the rat model of ASD than in the control group, which affects behavioural performance and social interaction patterns (Cheng et al., 2023). Phenylalanine can be converted to tyrosine, which is used to synthesise dopamine and serotonin. Reduced tyrosine levels in patients with ASD cause a reduction in dopamine levels, leading to distraction; while serotonin regulates the brain’s emotional, cognitive, and behavioural function (Wang et al., 2022). Excessive phenylalanine is neurotoxic and may manifest as developmental delay. It has been found that children with ASD consume high levels of phenylalanine in their diets, 25% of children with phenylketonuria have ASD, and a phenylalanine-free diet significantly improves ASD symptoms (Jennings and Basiri, 2022).

In addition, we found that the percentage of children with ASD who had sleep problems before rehabilitation was 73.08%%, and although it decreased after rehabilitation, it was still as high as 65%. This indicates that the prevalence of sleep problems in children with autism is high, which is consistent with the findings of Johnson and Zarrinnegar (2021). After the rehabilitation intervention, the quality of sleep in children with ASD improved, as evidenced by a statistically significant (*p* < 0.05) decrease in daytime sleepiness, night wakings and sleep onset delay. While these improvements suggest a positive impact of rehabilitation, the underlying mechanistic links between the gut microbiota and sleep in ASD require further clarification. Specifically, it remains unclear whether the rehabilitation intervention directly targets the microbiome to improve sleep, or whether microbiome changes occur as a consequence of improved sleep quality. One plausible mechanism is that rehabilitation promotes cognitive development and emotional regulation, which serves as a prerequisite for sleep improvement (Lou et al., 2022), subsequently reshaping the gut microbiota. Conversely, the intervention may directly modulate gut metabolic pathways (e.g., tryptophan metabolism) to facilitate sleep regulation. Sleep disturbances may exacerbate the manifestation of core symptoms of autism, such as repetitive locomotor and social deficits (Deng et al., 2022). Children with autism who experience insomnia are more likely to exhibit behavioral problems and reduced adaptive functioning (Fadini et al., 2015). Sleep problems in patients with ASD are closely associated with gut microbiota (Hua et al., 2020). In the present study, we found that *Lachnospiraceae_bacterium_Marseille_Q4251* was positively correlated with sleep duration based on the correlation analysis between CSHQ scores and differentially abundant species. Sleep-disordered breathing was positively correlated with *Clostridiaceae_bacterium_Marseille_Q4149* and *Dorea_sp_AF24_7LB*, whereas it showed a negative correlation with *Lachnospiraceae_bacterium_Marseille_Q4251* (all *p* < 0.05). These species belong to the family Lachnospiraceae, which has been reported to be closely related to sleep patterns, including associations with shorter nocturnal sleep duration and lower sleep efficiency (Wang et al., 2022). Maternal inflammation has been shown to program offspring’s microbiome in ways that persist into childhood and may predispose to neurodevelopmental sequelae (Vuong et al., 2020). Furthermore, the characteristic dietary restrictions common in children with ASD can directly and rapidly alter gut microbial composition and function, potentially exacerbating or maintaining a dysbiotic state (Yap et al., 2021). Therefore, the microbiota and metabolite profiles observed here could represent a functional interface through which these early-life factors influence neurodevelopment. Future longitudinal studies integrating maternal history, detailed dietary logs, and multi-omics profiling are needed to disentangle their specific contributions.

Our study combined the GDS-C scale with macrogenomics and metabolomics, exploring the correlation between growth and development and intestinal microecology in children with ASD. In addition, our study was dynamic, comparing the changes before and after 6-month rehabilitation training in children with ASD, which was more convincing.

## 5 Study limitations

This study initially enrolled 26 children with ASD, but 6 participants (23% attrition rate) were lost to follow-up, resulting in only 20 children with autism spectrum disorder completing the study. While this study did not conduct *a priori* sample size calculation, the sample size aligns with the characteristics of exploratory real-world research. For reference, similar studies on ASD microbiota have employed sample sizes of 24 ASD children and 49 healthy control children (Noone et al., 2025). This small sample size significantly reduced statistical power and increased the risk of type II errors (false negatives). The lack of significant differences in many between-group comparisons may likely be attributed to an insufficient sample size. And then, dietary patterns have a substantial impact on microbial composition; although we attempted to standardize dietary advice, the lack of detailed dietary intake records represents a potential confounding factor that should be addressed in future research. In addition, gastrointestinal pathology (e.g., constipation and diarrhea) is highly prevalent in ASD and can significantly influence the microbiome; future studies should control for these factors as covariates to isolate ASD-specific effects. Finally, the specific geographic and demographic characteristics of the cohort necessitate caution when extrapolating these results to children with different ethnic or regional backgrounds. Furthermore, although healthy controls were included to establish microbiome and metabolome baselines, clinical assessments (CSHQ and GDS-C) were not conducted for the control group. This precludes a direct comparison of post-intervention clinical recovery levels with typically developing children.

## 6 Conclusion

Rehabilitation intervention in children with autism spectrum disorder (ASD) improved sleep quality and specific developmental abilities. Multi-omics analyses identified alterations in gut microbial composition and metabolite profiles, including *Intestinibacter_bartlettii*, ornithine, and tyrosine, which were associated with clinical outcomes. These findings highlight potential mechanistic links between gut microbiota-mediated amino acid metabolism and sleep and developmental performance in ASD. The results support the use of key metabolites as biomarkers for monitoring rehabilitation effects. Future studies with larger cohorts and functional validation are warranted to confirm these observations and elucidate underlying mechanisms.

## Data Availability

The original contributions presented in the study are included in the article/supplementary material, further inquiries can be directed to the corresponding author.
